# Cancer stem cells from peritumoral tissue of glioblastoma multiforme: the possible missing link between tumor development and progression

**DOI:** 10.18632/oncotarget.25565

**Published:** 2018-06-15

**Authors:** Cristiana Angelucci, Alessio D’Alessio, Gina Lama, Elena Binda, Annunziato Mangiola, Angelo L. Vescovi, Gabriella Proietti, Laura Masuelli, Roberto Bei, Barbara Fazi, Silvia Anna Ciafrè, Gigliola Sica

**Affiliations:** ^1^ Istituto di Istologia ed Embriologia, Università Cattolica del Sacro Cuore-Fondazione Policlinico Universitario Agostino Gemelli, Rome, Italy; ^2^ Cancer Stem Cells Unit, IRCSS Casa Sollievo della Sofferenza, ISBReMIT-Institute for Stem Cell Biology, Regenerative Medicine and Innovative Therapies, Opera di San Pio da Pietrelcina, S. Giovanni Rotondo, Foggia, Italy; ^3^ Istituto di Neurochirurgia, Università Cattolica del Sacro Cuore–Fondazione Policlinico Universitario Agostino Gemelli, Rome, Italy; ^4^ Department of Biotechnology and Biosciences, University of Milan Bicocca, Milan, Italy; ^5^ IRCSS Casa Sollievo della Sofferenza, ISBReMIT-Institute for Stem Cell Biology, Regenerative Medicine and Innovative Therapies, Opera di San Pio da Pietrelcina, S. Giovanni Rotondo, Foggia, Italy; ^6^ Hyperstem SA, Lugano, Switzerland; ^7^ Department of Experimental Medicine, Sapienza University of Rome, Rome, Italy; ^8^ Department of Clinical Sciences and Translational Medicine, University of Rome Tor Vergata, Rome, Italy; ^9^ Department of Biomedicine and Prevention, University of Rome Tor Vergata, Rome, Italy

**Keywords:** glioblastoma cancer stem cells, peritumoral cancer stem cells, stemness markers, proliferation and invasiveness markers, H19 lncRNA and miR-675-5p

## Abstract

In glioblastoma multiforme (GBM), cancer stem cells (CSCs) are thought to be responsible for gliomagenesis, resistance to treatment and recurrence. Unfortunately, the prognosis for GBM remains poor and recurrence frequently occurs in the peritumoral tissue within 2 cm from the tumor edge. In this area, a population of CSCs has been demonstrated which may recapitulate the tumor after surgical resection. In the present study, we aimed to characterize CSCs derived from both peritumoral tissue (PCSCs) and GBM (GCSCs) in order to deepen their significance in GBM development and progression. The stemness of PCSC/GCSC pairs obtained from four human GBM surgical specimens was investigated by comparing the expression of specific stem cell markers such as Nestin, Musashi-1 and SOX2. In addition, the growth rate, the ultrastructural features and the expression of other molecules such as c-Met, pMet and MAP kinases, involved in cell migration/invasion, maintenance of tumor stemness and/or resistance to treatments were evaluated. Since it has been recently demonstrated the involvement of the long non-coding RNAs (lncRNAs) in the progression of gliomas, the expression of H19 lncRNA, as well as of one of its two mature products miR-675-5p was evaluated in neurospheres. Our results show significant differences between GCSCs and PCSCs in terms of proliferation, ultrastructural peculiarities and, at a lower extent, stemness profile. These differences might be important in view of their potential role as a therapeutic target.

## INTRODUCTION

It is currently believed that many solid tumors, including glioblastoma multiforme (GBM), develop from a small subset of cells with stem cell properties, termed “cancer stem cells” (CSCs) [1]. These cells reside in an aberrant niche that contains a variety of different stromal cells such as mesenchymal and immune cells, and presents a complex architecture with abnormal extracellular matrix components and an atypical vascular network [2]. Likewise normal stem cells, GBM CSCs show self-renewal, high proliferative potential, as well as the ability to differentiate into various tumor cell types [3]. From a clinical point of view, one of the main concerns with CSCs is their resistance to common chemo-radiotherapy, that can favor tumor recurrence [4, 5].

Although the administration of temozolomide has significantly improved patients’ overall survival [6], their outcome remains very poor with a life expectancy ranging from 14.6 to 16.7 months [7]. Due to the central role of vascularization in GBM progression, additional anti-angiogenic targeted therapies have been attempted without resulting in any significant improvement [8, 9]. In the absence of effective therapies, about 90% of patients show a high recurrence rate within 2 cm from tumor edge [10, 11], suggesting that the proximity to the tumor microenvironment most likely causes alterations in the peritumoral area and local relapse [12–14]. Therefore, the molecular and functional characterization of GBM neighboring tissue has raised great interest as a potential therapeutic target, although, data are still sparse compared to the amount of reports concerning tumor tissue [15]. In our previous studies, the expression of phosphorylated extracellular signal-regulated kinases 1/2 (pERK1/2), phosphorylated c-Jun NH2-terminal kinases (pJNK), stem cell markers such as Nestin, CD133, GD3 ganglioside and NG2 proteoglycan, as well as angiogenesis-related factors (CD105, VEGF, VEGFR1/2, HIF-1α and HIF-2α), has been demonstrated in both GBM and peritumoral tissue [16–21]. Interestingly, in the latter area, the above markers were expressed not only in cancer cells or reactive astrocytes, but also in apparently normal cells, even in the absence of frankly neoplastic cells, which suggests ongoing malignant transformation events.

The presence of elements with stem cell features in such an altered microenvironment might support their identity as CSCs, the most suitable candidates for the recurrence. Putative CSCs isolated from different regions of the same GBM or from the tumor mass and its neighboring tissues have been already reported to bear different characteristics in terms of growth properties, clonogenic potential, genetic abnormalities and *in vivo* tumorigenicity [22–24, 19]. In this framework, the present study aims to improve the characterization of CSCs obtained from GBM peritumoral tissue macroscopically devoid of neoplastic cells (PCSCs), by comparing their molecular profile and structural features to those derived from the tumor mass (GCSCs) [19]. In particular, the expression of stem cell markers (Nestin, Musashi-1 and SOX2), c-Met and its activated form pMet, pERK1/2, pJNK, H19 lncRNA and its encoded miR-675-5p, as well as the growth and ultrastructural characteristics of both GCSCs and PCSCs, were investigated. Nestin is a protein belonging to class VI of intermediate filaments, expressed during nervous system development and in adult stem and progenitor cells [25]. In GBM Nestin appears related to tumor cell dedifferentiation, invasiveness and malignancy [26–28]. Nestin knockdown in human GBM cell lines suppresses proliferation, migration and invasion, and increases F-actin expression and cell adhesion to the extracellular matrix [29]. Musashi-1 is a highly conserved RNA-binding protein with an essential role in stem cell phenotype maintenance and nervous system development. The expression of Musashi-1 is restricted to embryonic development and adult stem and progenitor cells but its overexpression occurs in tumors where it induces cell proliferation, differentiation arrest, apoptosis inhibition and allows self-renewal and pluripotency maintenance [30]. Together with Nestin and Musashi-1, SOX2, a nuclear transcription factor belonging to the SOX family, represents a master regulator of pluripotency and controls a variety of genes involved in the maintenance of the undifferentiated state during embryogenesis. In adults, SOX2 is re-expressed in cancer cells, particularly in the early stages of tumor development, suggesting its involvement in tumor-initiating events [31]. The maintenance of tumor stemness in GBM CSCs has been also recently attributed to the activation of c-Met, the tyrosine kinase receptor of the hepatocyte growth factor/scatter factor (HGF/SF), which also seems to mediate the acquisition of GBM CSCs radiotherapy resistance [32]. Moreover, the activation of extracellular signal-regulated kinases (ERK1/2) signaling can drive the expansion of CSC population and/or its innate radio-resistance in different tumors [33, 34]. Mitogen-activated protein kinases (MAPK)-ERK1/2, as well as JNK pathways, are essential for the stem cell-like properties of GBM CSCs [35, 36]. Moreover, Sunayama *et al*. reported that the concurrent inhibition of PI3K/AKT/mTOR and MAPK/ERK pathways effectively promotes GCSC differentiation and thereby suppresses their tumorigenicity [37]. Recent findings, suggesting the key role of endogenous cellular long noncoding RNAs (lncRNAs) in cancer progression, encouraged us to focus on H19 lncRNA, that has been found highly expressed in placenta, embryonic/fetal tissues and in several tumors including GBM [38, 39]. One of the two mature forms of miRNA generated from H19 lncRNA is miR-675-5p. H19 lncRNA and its derivate miR-675-5p positively correlate with glioma grade and the H19/miR-675 signaling axis has been recently reported to promote glioma cell invasion [40]. In our study, a comparative analysis of the expression of all the above-described molecules as well as of cell growth and structural features was performed on GCSC/PCSC pairs derived from four patients affected by primary GBM. Even though a high heterogeneity in the expression of stemness markers was found between PCSC/GCSC pairs, the results obtained suggest that PCSCs have a less malignant phenotype with respect to GCSCs, lower proliferative activity and some ultrastructural features related to a lower invasiveness. This study provides new insights into the biology of PCSCs, which may help in defining their potential role in GBM progression and improve patients’ treatment.

## RESULTS

### Morphology and proliferative activity of GCSCs and PCSCs

**Figure 1:**
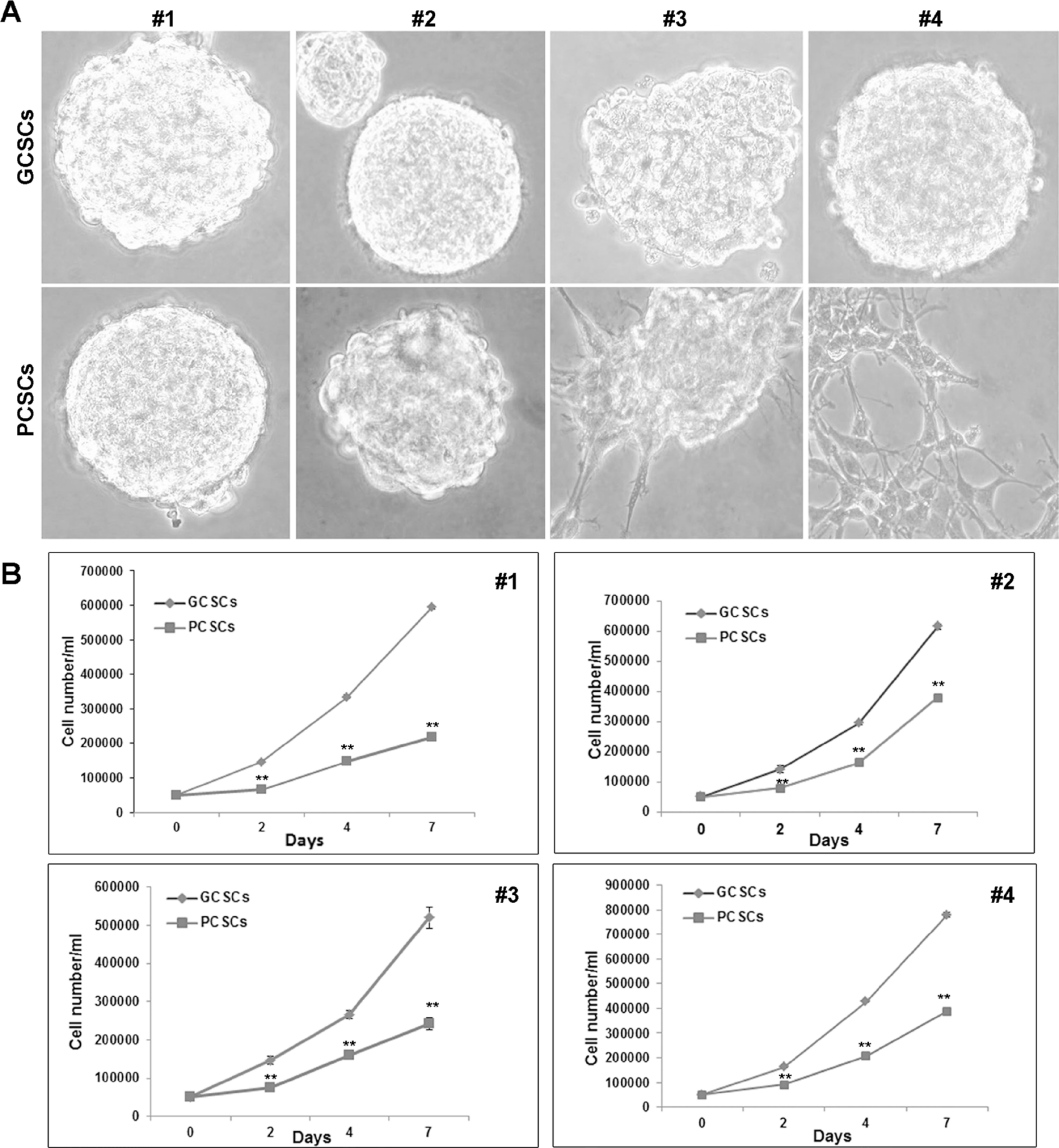
Morphological and proliferation analysis of GCSC/PCSC pairs (**A**) GCSCs derived from all the four patients, as well as PCSCs obtained from patients #1 and #2, grew as floating neurospheres. PCSCs corresponding to patients #3 and #4 grew as semi-adherent cells. Original magnification, ×400. (**B**) In each GCSC/PCSC pair (#1–4) analyzed, GCSCs (rumble) show a higher proliferation rate if compared to PCSCs (square). Values represent the mean ± SD of three independent experiments. Data were analyzed by Student *t* test, ^**^p < 0.001 vs GCSCs.

The morphology of neurospheres was observed by a phase-contrast microscope. GCSC and PCSC neurospheres displayed a bright surface and some of them showed a well-defined spherical contour, whereas others showed an irregular surface. In addition, while GCSCs obtained from all the four patients appeared, as expected, as free-floating neurospheres, PCSCs from patient #3 and #4 were semi-adherent (Figure 1A), indicating a certain heterogeneity among pairs. The proliferative capability of both cell populations was investigated at 2, 4 and 7 days in culture. The results of this analysis indicated a higher proliferation rate of GCSCs compared to PCSCs (*p* < 0.001) (Figure 1B).

### Stemness markers, c-Met, ERK1/2, JNK, H19 lncRNA and miR675-5p expression

#### Nestin expression

**Figure 2:**
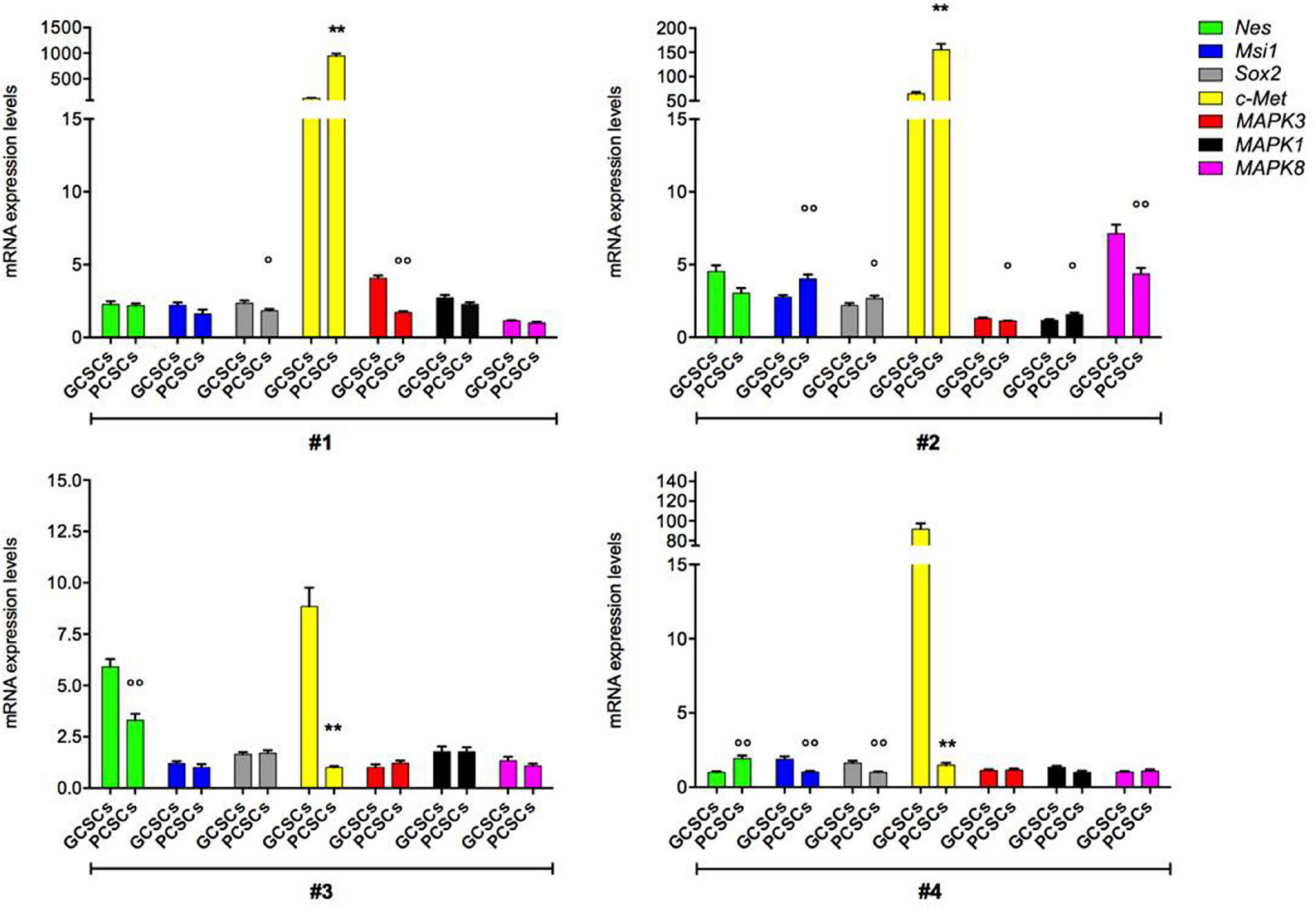
*Nes, Msi1, Sox2, c-Met, MAPK3, MAPK1 and MAPK8* gene expression in GCSC/PCSC pairs The expression level of the indicated genes was evaluated by qPCR in GCSCs and PCSCs. The relative RNA quantity was normalized to *GAPDH* endogenous control. *Nes* (gene coding for Nestin protein), *Msi1* (gene coding for Musashi-1 protein), *Sox2* (gene coding for SOX2 protein), *c-Met* (gene coding for c-Met protein), *MAPK3* (gene coding for ERK1 protein), *MAPK1* (gene coding for ERK2 protein) and *MAPK8* (gene coding for JNK protein). Bar graph show mean ± SE from three independent experiments. Data were analyzed by *t*-test pooled variance, ^°^*p* < 0.05, ^°°^*p* < 0.01 and ^**^p < 0.001 vs GCSCs.

**Figure 3:**
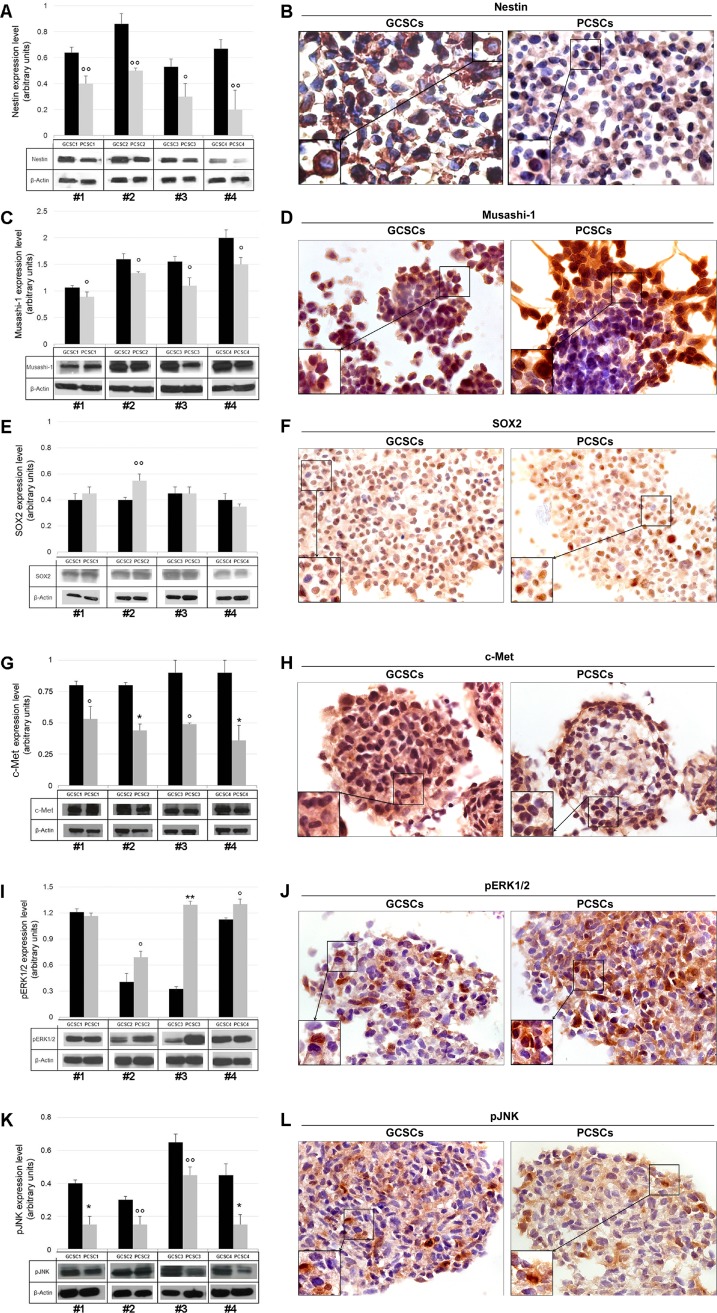
Nestin, Musashi-1, SOX2, c-Met, pERK1/2 and pJNK protein expression in GCSC/PCSC pairs Nestin (**A**), Musashi-1 (**C**), SOX2 (**E**), c-Met (**G**), pERK1/2 (**I**) and pJNK (**K**) protein expression was evaluated by Western blot analysis. Representative blots from three separate experiments yielding similar results are shown. The intensity of bands was quantified by densitometric analysis and normalized to those of β-actin (used as loading control). The values shown represent the mean ± SD of three independent experiments. Data were analyzed by Student *t*-test, ^°^*p* < 0.05, ^°°^*p* < 0.01, ^*^p < 0.005 and ^**^p < 0.001 vs GCSCs. Immunocytochemical analysis of Nestin (**B**), Musashi-1 (**D**) and SOX2 (**F**), c-Met (**H**), pERK1/2 (**J**) and pJNK (**L**) performed on GCSC and PCSC frozen sections (10 μm). All the investigated markers were expressed in both cell populations. Nestin immunoreactivity was found at the level of cell cytoplasm (B). Musashi-1 specific staining was seen in both the cytoplasm and the nucleus (D). SOX2 staining was mainly detected in the nucleus (F). c-Met (H), pERK1/2 (J) and pJNK (L) immunoreactivity was present at the level of both the cytoplasm and the nucleus. Original magnification, ×630. Details of immuno-positive and -negative cells are shown at higher magnification in the bottom left insets (B, D, F, H, J, L).

In order to assess the stemness profile of GCSCs and PCSCs, we evaluated the expression of the intermediate filament protein Nestin. Our analysis revealed that Nestin coding gene (*Nes*) was expressed at a different extent in both GCSCs and PCSCs (Figure 2). In particular, in patient #3, the *Nes* level was lower in PCSCs than in GCSCs (*p* < 0.001), whereas in patient #4 a higher *Nes* expression was observed in PCSCs (*p* < 0.001). No significant difference in Nestin mRNA level was found between PCSCs and GCSCs of patient #1 and #2. With respect to the heterogeneity seen in *Nes* gene expression, Western blot analysis demonstrated lower levels of Nestin protein in all PCSCs compared to GCSCs (Figure 3A; *p* < 0.05, *p* < 0.01). Immunohistochemical analysis showed a diffuse Nestin staining in the cytoplasm of both cell types (Figure 3B).

#### Musashi-1 expression

Due to its role in the maintenance of the stem-cell state, Musashi-1 gene (*Msi1*) and protein expression were analyzed. Similarly to *Nes*, *Msi1*, although detected in all pairs of CSCs, showed a heterogeneous pattern of expression among the different PCSC/GCSC pairs (Figure 2), while Musashi-1 protein levels resulted lower in all PCSCs compared to GCSCs (Figure 3C; *p* < 0.05). As shown in Figure 3D, immunoreactivity of Musashi-1 protein was detected in both cytoplasmic and nuclear compartment of both GCSCs and PCSCs.

#### SOX2 expression

The expression of the stem cell marker SOX2 was evaluated at the mRNA and protein level. *Sox2* gene expression did not display a definite trend among the different GCSC/PCSC pairs (Figure 2). Western blot analysis demonstrated a comparable level of SOX2 protein in all samples of GCSCs and PCSCs, with the only exception of patient #2 where it was higher in PCSCs (Figure 3E; *p* < 0.01). SOX2 immunolabeling was restricted into the nucleus in both cell types, indicating the activated status of the protein (Figure 3F).

#### c-Met expression

The analysis of the receptor tyrosine kinase c-Met expression by qPCR demonstrated that *c-Met* gene level in PCSCs of patients #1 and #2 was higher than in GCSCs (Figure 2, *p* < 0.001). In patients #3 and #4 *c-Met* expression was lower in PCSCs (Figure 2; *p* < 0.001). As for c-Met protein expression, similarly to what was described for Nestin and Musashi-1, it was significantly lower in PCSCs compared to GCSCs (Figure 3G; *p* < 0.05 and *p* < 0.005). However, none of the four CSC pairs analyzed showed detectable level of the activated form of c-Met (pMet; data not shown). The c-Met expression was widely detected in both PCSC and GCSC populations (Figure 3H) and the immunoreactivity was mainly localized in both the cytoplasm and the nucleus.

#### ERK1/2 and JNK expression

As regards ERK1/2 and JNK expression, our data indicated lower levels of *MAPK3* gene (coding for ERK1 protein) in PCSCs of patients #1 and #2 (*p* < 0.01 and *p* < 0.05, respectively), while no significant difference was observed between GCSCs and PCSCs of patients #3 and #4 (Figure 2). No significant variations were detected in the transcript levels of *MAPK1* (coding for ERK2 protein) and *MAPK8* (coding for JNK protein), except for one patient (#2), among GCSC and PCSC pairs (Figure 2).

As for the expression of the phosphorylated (p) form of ERK1/2 and JNK, Western blot analysis demonstrated that PCSCs displayed levels of pERK1/2 higher than GCSCs in three out of four neurosphere pairs (Figure 3I). On the contrary, as regards pJNK, the protein level was consistent with the expression of most of the stem cell markers analyzed, being lower in PCSCs (Figure 3K). The apparent discrepancy between pERK1/2 and pJNK profile expression might be due to multiple mechanisms of MAPK activation [41]. Immunocytochemical analysis showed the presence of both pERK1/2 and pJNK in the cytoplasm as well as in some of the nuclei of GCSCs and PCSCs (Figure 3J, 3L).

#### H19 lncRNA and miR675-5p expression

**Figure 4:**
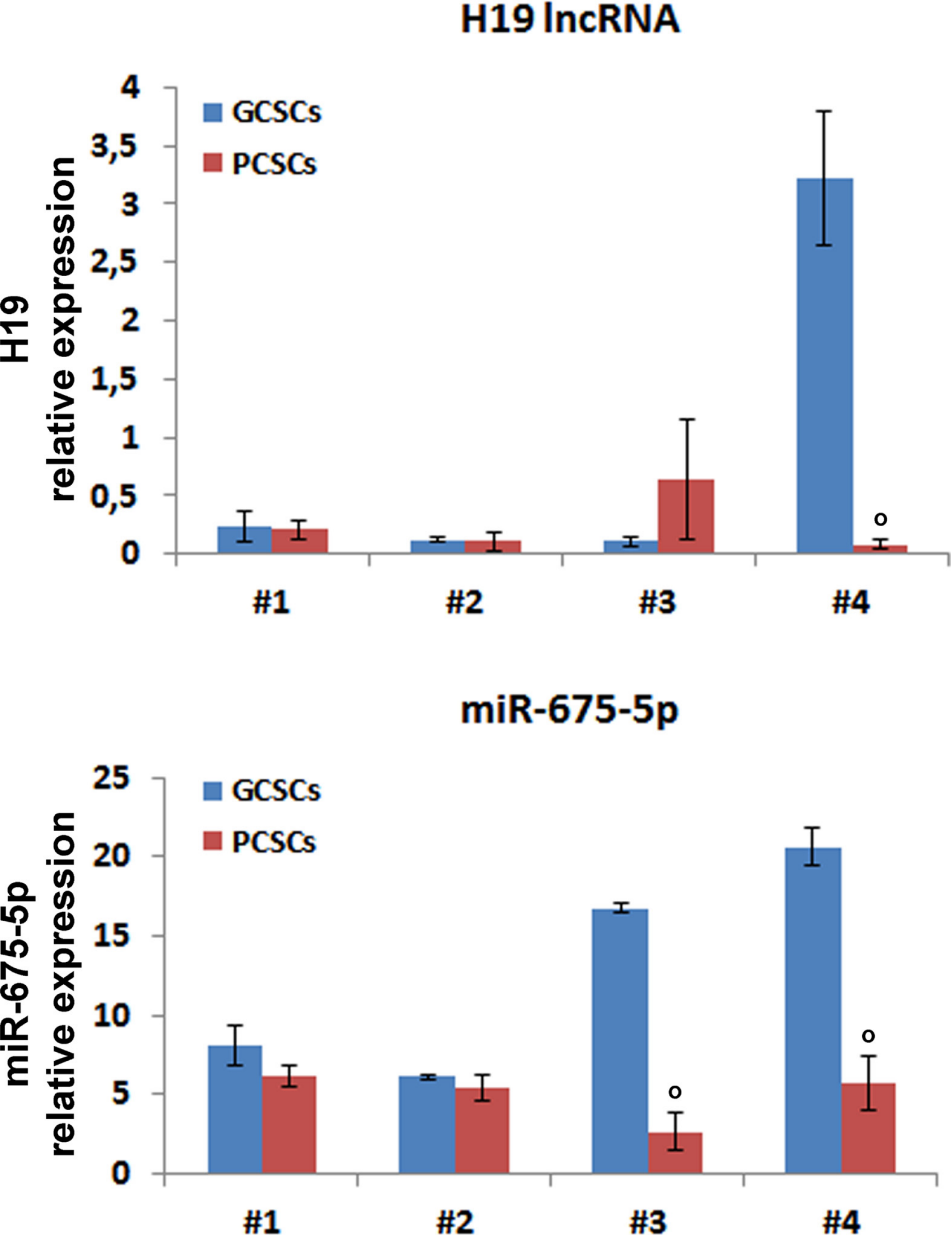
H19 lncRNA and miR-675-5p expression in GCSC/PCSC pairs The expression level of H19 lncRNA and miR-675-5p was evaluated by qPCR in GCSCs and PCSCs. All values were expressed as compared to those found in human astrocytes, where H19 expression was set = 1. Expression levels were normalized against U6 snRNA and TBP mRNA as controls for miR-675-5p or H19 lncRNA, respectively. The values shown represent the mean ± SD of two independent experiments. Data were analyzed by Student *t*-test; ^°^*p* < 0.05 vs GCSCs.

As for H19 lncRNA, no specific trend of expression was apparent in the GCSC/PCSC pairs (Figure 4). Indeed, no significant differences were observed in H19 lncRNA levels between PCSCs and GCSCs from patients #1–3, while it was clearly enriched in GCSCs obtained from patient #4. In addition, we measured the expression levels of miR-675-5p, the prevalent mature form of miR-675 (www.mirbase.org). MiR-675-5p expression in GCSC/PCSC pairs revealed the same trend as of H19 lncRNA in the neurosphere pairs derived from patients #1, #2 and #4, while in the GCSC/PCSC pair of patient #3 it did not mirror that of H19 lncRNA, being lower in PCSCs.

### Ultrastructural features

**Figure 5:**
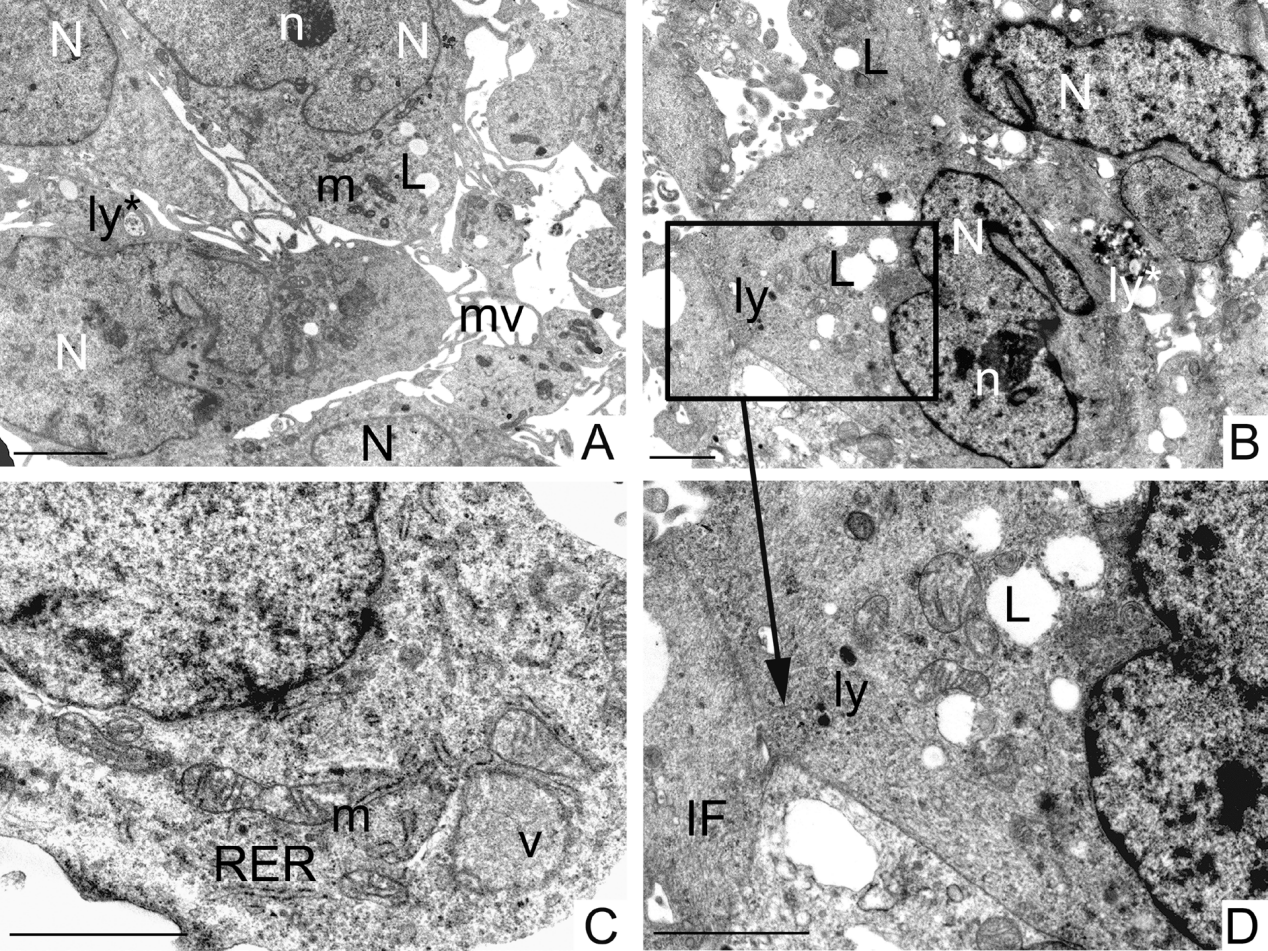
Ultrastructural analysis of GCSCs GCSCs showed irregular nuclei (N) mainly formed by euchromatin with marginated heterochromatin and prominent nucleoli (n) (**A**, **B**). Rough endoplasmic reticulum (RER), mitochondria (m), vesicles (v) and intermediate filaments (IF) were visible in the cytoplasm (**C**, **D**). Lipid droplets (L), primary and secondary lysosomes (ly and ly^*^, respectively) were observed (A, B and D). Plasma membranes showed microvilli (mv) (A) and loose cell-cell contacts (square in B and at higher magnification in D). Scale bars = 2 μm.

**Figure 6:**
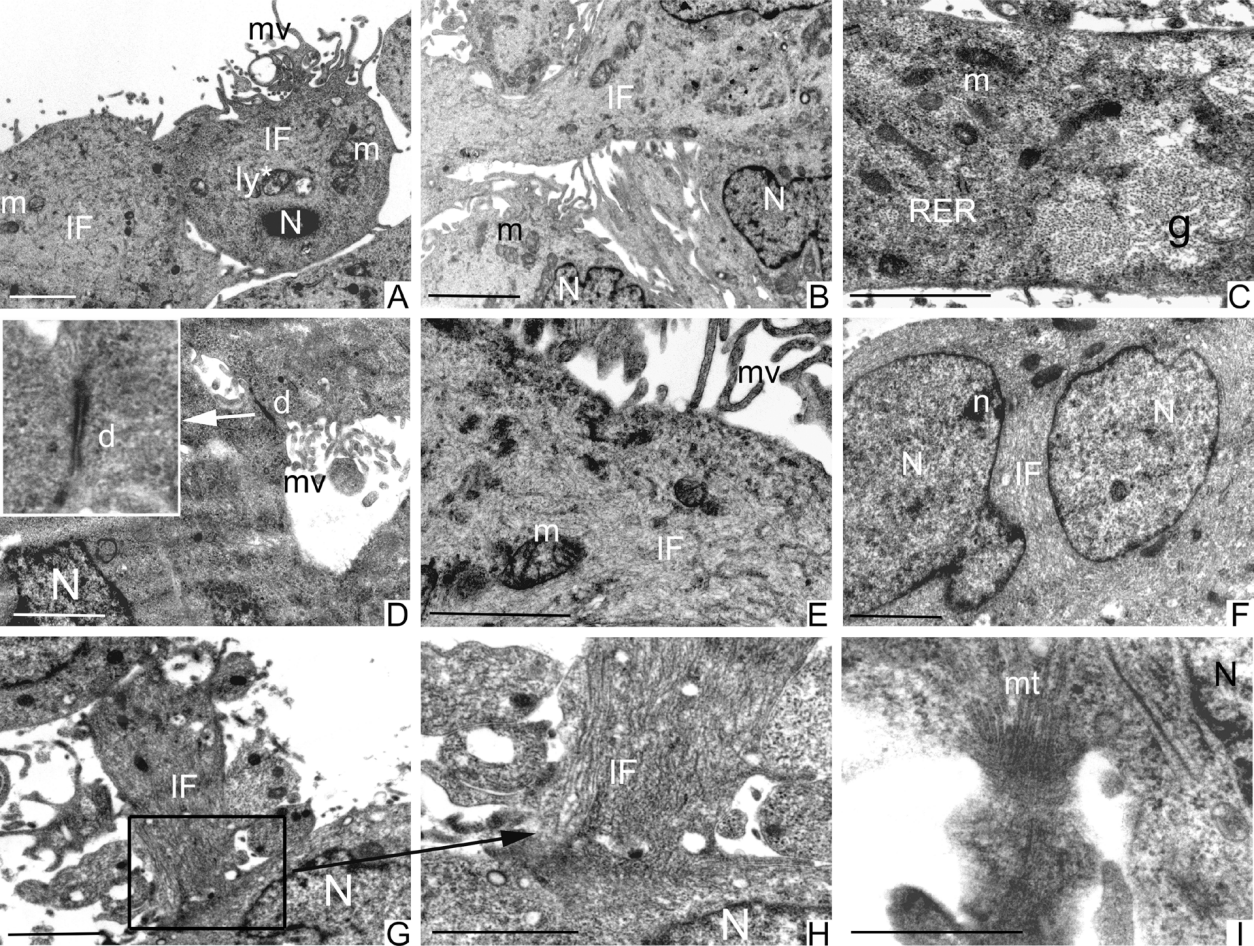
Ultrastructural analysis of PCSCs PCSCs showed irregular nuclei (N) (**A**, **B** and **F**) and sometimes multiple nuclei with evident nucleoli (n) (F). Mitochondria (m) were visible (A–**C**, **E**). Cells were particularly rich in intermediate filaments (IF) located in the cytoplasm and in the perinuclear region (A, B, E–H). Microvilli (mv) were present on the cellular membrane (A, **D** and E). Abundant glycogen (g) stored free in the cytoplasm (C) was noted. Fully organized desmosomes (d) were also visible (D). Loose cell-cell contacts were established by cellular processes rich in IF (square in **G** and at higher magnification in **H**) and microtubules (mt) (**I**). Scale bars = 2 μm.

TEM ultrastructural analysis of GCSCs (Figure 5) and PCSCs (Figure 6) was performed on neurospheres derived from biopsies of four different GBM patients. All examined cell cultures contained a phenotypically heterogeneous population and cells were generally arranged in rounded formations.

PCSCs and GCSCs shared several characteristics, such as irregular nuclei, mainly constituted by euchromatin with marginated heterochromatin (Figures 5A, 5B and 6A, 6B, 6F) and prominent nucleoli (Figures 5A, 5B and 6F). Rough endoplasmic reticulum, vesicles, intermediate filaments (Figures 5C, 5D and 6A–6C, 6E–6H) and mitochondria (Figures 5A, 5C and 6A–6C, 6E) were also visible in the cytoplasm. In addition, the plasma membranes of both PCSCs and GCSCs showed abundant microvilli (Figures 5A and 6A, 6D and 6E).

Lipid droplets as well as primary and secondary lysosomes were observed in GCSCs (Figure 5A, 5B, 5D). In particular, primary lysosomes are smaller and homogeneously electron dense while the secondary lysosomes are bigger and irregularly electron-dense because they contain digested material. The GCSC plasma membranes showed loose cell-cell contacts (Figure 5B, 5D); organized desmosomes or junctional complexes were not detected, while they were visible in PCSCs (Figure 6D). In addition, PCSCs were particularly rich in glycogen. Glycogen, stored free in the cytoplasm, was visible as electron-dense particles about the size of ribosomes (Figure 6C). Intermediate filaments were apparently more abundant in these latter neurospheres, if compared to GCSCs (Figure 6). In particular, intermediate filaments were variously arranged and located in the cytoplasm (Figure 6A, 6B, 6E, 6G and 6H) and in the perinuclear region (Figure 6F). In addition, PCSCs showed long processes supported by abundant microtubules (Figure 6I) and intermediate filaments. These processes established loose cell-cell contacts with neighbouring cells (Figure 6G–6I).

## DISCUSSION

It is now widely accepted that GBM, as it occurs in other tumors, is initiated and maintained by CSCs, referred to as a subset of cells with stem-like properties that display an unlimited proliferative capacity and ability to differentiate into the different cell types that compose the tumor mass. Due to their high resistance to conventional treatments [4, 5, 42] and despite the aggressive therapy commonly administered to patients, the presence of this cell population can account for the high frequency of recurrence seen in GBM-affected patients. To this regard, it is likely that CSCs are also present in the peritumoral tissue where they may function as a reservoir of cancer initiating cells after surgical resection. Previous reports have demonstrated that GBM induces alterations in the peritumoral tissue by producing soluble factors that contribute to the establishment of an aberrant niche that allows CSCs to survive and resist to common therapies [12–14]. The present study aimed to expand our previous findings [16–21] on the role of the peritumoral tissue during GBM progression by focusing on the molecular and structural characterization of CSCs residing in this area. Therefore, we investigated and compared in GCSCs and PCSCs the expression of stem cell markers as well as molecules involved in the maintenance of tumor stemness and/or resistance to treatments, the proliferative activity and the ultrastructural features. In agreement with findings by other authors [22–24] and with our previous observations [19], the results of this study support the existence of two different malignant populations with stem cell-like characteristics in GBM and in the neighboring tissue, respectively.

Here we report that both GCSCs and PCSCs derived from four patients express transcripts of Nestin, Musashi-1, SOX2, c-Met, MAPK1/3 and JNK. Nevertheless, it was not possible to draw a definite trend, due to the high heterogeneity in gene transcript levels shown by GCSCs and PCSCs isolated from different patients. This might be explained by the existence of various CSC subpopulations endowed with a different stemness/malignancy signature within the neurospheres derived from GBM and peritumoral tissue. This hypothesis may also support our findings on protein expression, which does not always match the mRNA levels. This is not a surprising event since various mechanisms may contribute to the protein synthesis rate and half-life [43]. However, the higher protein levels of both Nestin and Musashi-1, expressed by GCSCs, indicate a less differentiated as well as a more aggressive status of these CSCs compared to PCSCs. Concerning SOX2, which is thought to play a key role in the maintenance of GBM stem-like cell properties, we found an overall comparable protein level in GCSCs and PCSCs. This may be due to the existence of a specific CSC hierarchy which determines the timing of expression of stemness markers during stem cell neoplastic transformation [44]. Then, SOX2 may be expressed by CSCs in the early phases of their malignant transformation and its level might remain unchanged in GCSCs. Consistent with this hypothesis, it has been previously demonstrated that SOX2 mRNA expression was greater in lower grade gliomas [45].

Since the activation of c-Met in GBM has been demonstrated to be involved in the establishment of radiotherapy resistance as well as in the expansion and maintenance of stemness features of the CSC pool [32, 46], the higher levels of c-Met found in GBM CSCs were not surprising [47, 48]. As previously reported by others, we did not detect the expression of the activated form of c-Met (pMet) under basal growth conditions [49]. However, we found c-Met immunostaining into the nuclei that may indicate the presence of the C-fragment, produced by a proteolytic cleavage of c-Met, which is independent from the ligand activation. The nuclear C-fragment seems to act as a transcription factor of genes involved in migration and invasiveness [50]. However, the expression of c-Met in PCSCs confirms the malignant nature of the stem cells residing in the peritumoral area and may contribute to explain their radio-resistance. In addition, the expression of pERK1/2 and pJNK in GCSCs and mainly in PCSCs has particular relevance since these molecules are known to be altered in pathological conditions, in which they increase proliferation and promote migration and invasion. Indeed, Nakada and coworkers showed that the activation of ERK1/2 correlates with an overexpression of integrin α3 that may contribute to the invasive nature of CSCs in GBM [51]. Moreover, it has been reported that treatment with retinoic acid triggers differentiation and, in turn, reduces proliferation and self-renewal in GBM CSCs in an ERK1/2-dependent manner [52]. The JNK function in GBM biology is complex and needs to be fully defined. In agreement with our results, high JNK levels have been described in self-renewing GBM CSCs and its knockdown or pharmacological inhibition determined the reduction of sphere formation, clonogenicity and stem cell marker expression [53, 54]. These events indicate that the JNK signaling pathway represents an important mechanism in the maintenance of the stemness features of GBM CSCs. As for the above markers, the detection of activated JNK in PCSCs confirms their tumor stem cell identity. A role in GBM stem-like cell biology has also been recently reported for the H19 lncRNA [55]. The authors demonstrated that overexpression of H19 lncRNA in stem-like cells derived from two human GBM cell lines enhances their ability to form neurospheres, thus promoting their self-renewal, a hallmark of stem-like cells in GBM. In agreement with data indicating that miR-675-5p contributes to glioma cell invasion and positively correlates with glioma grade [40], we found a higher expression of the miR-675-5p in GCSCs of two out of four neurosphere pairs.

To the best of our knowledge, this is the first time that a detailed ultrastructural analysis of CSCs derived from peritumoral tissue has been performed and compared to that carried out on CSCs isolated from GBM. Here, we show that the two types of neurospheres share some features that include evident irregular nuclei, with prominent nucleoli indicating their malignancy, and well represented mitochondria, rough endoplasmic reticulum, vesicles and microtubules. Nevertheless, unlike PCSCs, which display fully organized desmosomes and junctional complexes, GCSCs were devoid of cell junctions and were characterized by loose cell-cell contacts, as also demonstrated by other authors [56]. These observations clearly indicate that GCSCs, in contrast to PCSCs, have an enhanced capability to migrate and invade the neighboring tissues. Noteworthy, microvilli occurrence on the surface of GCSCs and PCSCs may be related to an augmented resistance of these cells towards cytotoxic lymphocytes, in agreement with previous findings [57].

We previously demonstrated that PCSCs express a lower level of NG2 and Ki67 and induced, in immunocompromised mice, intracranial tumors which were less vascularized if compared with those induced by GCSCs and with a proliferative potential similar to grade III gliomas [19]. The present study demonstrate that CSCs residing in the peritumoral tissue show different behavior with respect to those present in GBM. In fact, the comparison between GCSCs and PCSCs has highlighted differences in term of proliferative potential, ultrastructure and expression of stem cell markers, c-Met, MAPK, H19 lncRNA and miR-675-5p, confirming that PCSCs are less aggressive. All the emerged features, taken as a whole, provide new insights into the cellular and molecular properties of PCSCs, that may be helpful in view of finding a suitable treatment and should lead to a deeper knowledge of GBM biology and progression.

## MATERIALS AND METHODS

### Neurosurgical procedure for tumor debulking

Tumor debulking was performed in four patients affected by primary supratentorial GBM who did not receive any therapy before surgery. A very careful neurosurgical procedure using simultaneously two different computerized navigational systems was applied: the SteathStation^®^ Navigational System (Medtronic, USA) and the MyLabXGV70^®^ equipped with Virtual Navigator^®^ (Esaote, Italy), which combine pre-surgery MR images of the patient with real-time ultrasound scan images acquired during surgery. The procedure allows for the identification of the necrotic areas of the tumors, non-necrotic core areas and peritumoral areas, which were sampled out separately. The two latter areas used in our experiments. The method is rather effective and we have previously reported data obtained using this technique that showed the presence of CSC subtypes in human GBM [19, 22, 58]. The protocol was approved by the Ethics Committee of the “Università Cattolica del Sacro Cuore”, Rome, Italy (Prot. A/205/2011) and all patients gave their informed consent prior to surgery.

### Primary cultures and culture propagation

From each of the four patients, paired tissue specimens were obtained from the tumor without necrotic areas (GBM) and from the white matter at a distance ≤1 cm from the macroscopic tumor border, where neoplastic-appearing cells were not observed (peritumoral tissue). As previously reported, the two areas were sampled separately, analyzed by an expert pathologist according to the World Health Organization guidelines and used in our experiments. Tissues (fresh surgical specimens) were dissected out, cut with scissors into 1 mm^3^ sections, then incubated and digested in a solution containing papain (24.5 U/mg; Worthington Biochemical Corp, Lakewood, NJ, USA) under continuous oxygenation for 45 min at 37° C [58, 59].

Primary cultures were then collected by centrifugation and plated in serum-free NeuroCult^®^ NS-A Basal Medium with NeuroCult^®^ NS-A Proliferation Supplement, 20 ng/ml epidermal growth factor, 10 ng/ml basic fibroblast growth factor and 0.2% heparin (StemCell Technologies, Vancouver, BC, Canada) [19, 58, 59]. The cells isolated from tumor (GCSCs) and peritumoral (PCSCs) tissues generated neurospheres showing the typical properties of cancer stem-like cells as self-renewal, pluripotency, capability to establish a long-term expanding cultures as well as to generate tumor when inoculated into the right striatum of Scid/bg mice [19]. For propagation, GCSC and PCSC neurospheres were mechanically dissociated and cultured in the above described medium [19, 58, 59]. In all the experiments, GCSCs and PCSCs were used between passages 15 and 20. Normal human astrocytes (SC-1800), used as positive control in the experiments evaluating H19 lncRNA/miR-675-5p expression, were obtained from CliniSciences (Guidonia Montecelio, Italy) and cultured in astrocyte growth medium Kit (SC-1801, CliniSciences, Italy).

### GCSC and PCSC proliferation analysis

Growth curves were plotted to compare the GCSC and PCSC proliferation rate. Cells were plated at a density of 8,000 cells/cm^2^ in culture medium. Every two days for one week, the two cell populations were collected, mechanically dissociated into single cell suspensions and counted with a hemocytometer.

Determination of cell viability by trypan blue dye exclusion test was carried out and all GCSC/PCSC pairs showed a viability never <90%.

### RNA extraction and quantitative real-time PCR (qPCR)

Total RNA from GCSCs and PCSCs was extracted using the RNeasy Mini Kit (Qiagen, Chatsworth, CA, USA). cDNA was obtained by using SuperScript III Reverse Transcriptase (RT) (Invitrogen) and 1 μg of total RNA was primed with oligo-dT for cDNA synthesis. Quantitative PCR reactions were run in duplicate using IQTM SYBR Green QPCR Supermix (Bio-Rad) and fluorescent emission was recorded in real-time (Chromo 4 Four-Color Real-Time PCR Detector, Bio-Rad). Gene expression profiling was completed using the comparative Ct method of relative quantification. Relative RNA quantities were normalized to GAPDH as endogenous control. To evaluate the expression of *Nes* (gene coding for Nestin protein), *Msi1* (gene coding for Musashi-1 protein), *Sox2* (gene coding for SOX2 protein), *c-Met* (gene coding for c-Met protein)*, MAPK3* (gene coding for ERK1 protein), *MAPK1* (gene coding for ERK2 protein) and *MAPK8* (gene coding for JNK protein), the following primer pairs were employed: *MAPK1F1* (5′-GATCACACAGGGTTCCTGAC-3′) and *MAPK1R1* (5′-CAGAATGCAGCCTACAGACC-3′); *MAPK3F1* (5′-CTTCCTGACGGAGTATGTGG-3′) and *MAPK3R1* (5′-CCGGTTAGAGAGCATCTCAG-3′); *MAPK8F1* (5′-GTGTCTCCCTGGACTGTGAC-3′) and *MAPK8R1* (5′-GAAGGAGAGGAATGGAGGAG-3′); *MusashiF1* (5′-CAAAGTGTCTATCTGGGTGTGG-3′) and *MusashiR1* (5′-TATGATACAGGACGGGATGG-3′); Sox2F1 (5′-CCCTGTGGTTACTCTTCCT-3′) and *Sox2R1* (5′-GTAGTGCTGGGACATGTGAAGT-3′); *c-MetF1* (5′-TCCAAATATTGCCGTTTCATA-3′) and *c-MetR1* (5′-CTATTGATGCGTTCATGCTCT-3′); *NestinF1* (5′-TTGCAGATGAGGAAGAAAGTG-3′) and *NestinR1* (5′-CACAGAATCAGACTCCAGGAA-3′). Total RNA for H19 and miR-675-5p was isolated by TRIZOL reagent (Thermo Fisher Scientific), following manufacturer's instructions. For H19 RNA measurement, 0.5 μg of total RNA isolated from samples (pre-treated with DNase I, New England Biolabs) were used to generate cDNA by the SensiFAST cDNA Synthesis Kit (Bioline, London, UK), according to the manufacturer's instructions. For miR-675-5p measurement, 10 ng of total RNA were used to generate cDNA by TaqMan MicroRNA Reverse Transcription Kit (Applied Biosystems-Life Technologies), according to the manufacturer's instructions. Quantitative PCRs were conducted on an StepOnePlus Real-Time PCR System (Applied Biosystems-Life Technologies) using TaqMan Universal PCR Master Mix and the specific TaqMan^®^ Assays (probe and primer sets) (Applied Biosystems-Life Technologies). The small endogenous nuclear TBP and RNA U6 (RNU6B) were used as controls for normalization of H19 lncRNA and miR-675-5p, respectively. The relative amount of each substrate was calculated by the 2^−ΔΔCT^ method [60], and all results were expressed as compared to H19 lncRNA or miR-675-5p expression found in normal human astrocytes [61] set as = 1. All the primers were supplied by Applied Biosystems: RNU6B, ID 001093; TBP, ID Hs99999910_m1; H19, ID Hs00262142_g1; miR-675-5p, ID 002005.2.4.

### Western blot analysis

GCSCs and PCSCs were lysed in cell lysis buffer (150 mM NaCl, 1% Nonidet P-40, 0.5% Triton X-100, 0.5 mM EDTA, 0.1% SDS, 50 mM Tris-HCl, pH 7.6) containing a protease and phosphatase inhibitor cocktail (Sigma-Aldrich, St. Louis, MO, USA) supplemented with 17.4 μg/ml PMSF (Sigma-Aldrich). Proteins were quantified by Bradford Protein Assay (*Bio-Rad* Laboratories, Inc., Hercules, CA, USA). Twenty-five μg of total proteins were fractioned by 8–10% SDS-PAGE, transferred electrophoretically to a PVDF membrane (Immobilon-P Transfer Membrane, Millipore, Bedford, MA, USA) and immunoblotted with primary anti-Nestin (Clone 10C2, diluted 1:1000, Millipore), anti-Musashi-1 (clone 282613, diluted 1:500, R&D Systems, Inc.), anti-SOX2 (Clone 245610, diluted 1:500, R&D Systems), anti-c-Met (C-12, diluted 1:500, Santa Cruz Biotechnology), anti-phospho-Met (Tyr1234/1235) and anti-phospho-Met (Tyr 1349) (diluted 1:1000, Cell Signaling Technology), anti-phospho-pERK1/2 (Clone D13.14.4E, diluted 1:1000, Cell Signaling Technology), anti-pJNK (Clone G-7, diluted 1:500, Santa Cruz Biotechnology).

Membranes were then incubated with HRP-conjugated secondary antibodies (diluted 1:160000, Sigma-Aldrich). Detection of the bound antibody by enhanced chemiluminescence was performed using Immun-Star™ WesternC™ Chemiluminescence Kit (Bio-Rad Laboratories Inc, Hercules, CA, USA), according to the manufacturer's instructions. Membranes were reprobed with an anti-β-actin monoclonal antibody (clone AC15, diluted 1:5000, Sigma-Aldrich) as an internal control for protein loading. The signals were quantitated by densitometry (Chemi Doc Documentation System/Quantity One quantitation software, Bio-Rad). Densitometric units of the protein of interest were *normalized with* respect to total β-*actin* protein expression.

### Immunocytochemical analysis

GCSCs and PCSCs were fixed in 4% paraformaldehyde, cryoprotected in 30% sucrose, snap-frozen in liquid nitrogen and stored at –80° C until use. Immunocytochemistry was performed on frozen sections (10 μm) obtained from the neurospheres. Endogenous peroxidase activity was inhibited by incubation in 0.3% hydrogen peroxide and nonspecific reaction was blocked with Super Block, (Super Block, UCS Diagnostic S.r.l., Morlupo, Italy). The sections were then incubated overnight at 4° C with one of the following antibodies: anti-Nestin (clone 10C2, diluted 1:200, Millipore, Darmstadt, Germany), anti-Musashi-1 (Clone 282613, diluted 1:50, R&D Systems, Inc., Minneapolis, MN, USA), anti-SOX2 (Clone 245610, diluted 1:200, R&D Systems), anti-c-Met (C-12, diluted 1:300, Santa Cruz Biotechnology, Santa Cruz, CA, USA), anti-phospho-ERK1/2 (Clone E10, diluted 1:250, Cell Signaling Technology, Beverly, MA, USA) and anti-pJNK (Clone G-7, diluted 1:50, Santa Cruz Biotechnology) which recognize the JNK1, JNK2, and JNK3 isoforms. Next, the sections were incubated at room temperature with HRP/Fab polymer conjugate (SuperPicTure Polymer DetectionKit, Thermo Fisher Scientific Inc. Waltham, MA, USA). A peroxidase DAB substrate kit (Vector Laboratories Inc., Burlingame, CA, USA) was used to visualize the immunostaining and Mayer's hematoxylin was used to lightly counterstain nuclei. Negative controls were performed by omitting the primary antibodies.

### Ultrastructural analysis

GCSCs and PCSCs were fixed in 2.5% glutaraldehyde in PBS (pH 7.4) and processed for transmission electron microscopy (TEM), following routine procedures [62]. Briefly, cells were post-fixed with 1.33% osmium tetroxide, dehydrated in graded alcohols, and then embedded in Epon 812 resin (Fisher Chemical Co., Dallas, TX, USA). The resin was allowed to polymerize in a dry oven at 60° C, and specimens were cut on a Reichert ultra-microtome, stained with uranyl acetate and lead citrate, and observed under a Philips Morgagni 268D transmission electron microscope.

### Statistical analysis

Statistical analysis was performed using the GraphPad Prisma software (GraphPad Software, San Diego, CA, USA). Data derived from at least two experiments (run in triplicates) were reported as mean value ± SD or SE. The comparison between GCSC and PCSC mRNA level was performed by *t* test pooled variance. As for the protein expression analysis, H19 lncRNA and miR-675-5p as well as growth curves, the comparison between GCSCs and PCSCs was carried out by unpaired two-tailed Student *t*-test. An alpha level of less than 0.05 (*p* < 0.05) was considered statistically significant.
